# Genome-wide metabolic re-annotation of *Ashbya gossypii*: new insights into its metabolism through a comparative analysis with *Saccharomyces cerevisiae* and *Kluyveromyces lactis*

**DOI:** 10.1186/1471-2164-15-810

**Published:** 2014-09-24

**Authors:** Daniel Gomes, Tatiana Q Aguiar, Oscar Dias, Eugénio C Ferreira, Lucília Domingues, Isabel Rocha

**Affiliations:** CEB - Centre of Biological Engineering, Universidade do Minho, Campus de Gualtar, 4710-057 Braga, Portugal

**Keywords:** Genome re-annotation, *Ashbya gossypii*, Metabolic functions, Yeast metabolism

## Abstract

**Background:**

*Ashbya gossypii* is an industrially relevant microorganism traditionally used for riboflavin production. Despite the high gene homology and gene order conservation comparatively with *Saccharomyces cerevisiae*, it presents a lower level of genomic complexity. Its type of growth, placing it among filamentous fungi, questions how close it really is from the budding yeast, namely in terms of metabolism, therefore raising the need for an extensive and thorough study of its entire metabolism. This work reports the first manual enzymatic genome-wide re-annotation of *A. gossypii* as well as the first annotation of membrane transport proteins.

**Results:**

After applying a developed enzymatic re-annotation pipeline, 847 genes were assigned with metabolic functions. Comparatively to KEGG’s annotation, these data corrected the function for 14% of the common genes and increased the information for 52 genes, either completing existing partial EC numbers or adding new ones. Furthermore, 22 unreported enzymatic functions were found, corresponding to a significant increase in the knowledge of the metabolism of this organism. The information retrieved from the metabolic re-annotation and transport annotation was used for a comprehensive analysis of *A. gossypii*’s metabolism in comparison to the one of *S. cerevisiae* (post-WGD – whole genome duplication) and *Kluyveromyces lactis* (pre-WGD), suggesting some relevant differences in several parts of their metabolism, with the majority being found for the metabolism of purines, pyrimidines, nitrogen and lipids. A considerable number of enzymes were found exclusively in *A. gossypii* comparatively with *K. lactis* (90) and *S. cerevisiae* (13). In a similar way, 176 and 123 enzymatic functions were absent on *A. gossypii* comparatively to *K. lactis* and *S. cerevisiae*, respectively, confirming some of the well-known phenotypes of this organism.

**Conclusions:**

This high quality metabolic re-annotation, together with the first membrane transporters annotation and the metabolic comparative analysis, represents a new important tool for the study and better understanding of *A. gossypii*’s metabolism.

**Electronic supplementary material:**

The online version of this article (doi:10.1186/1471-2164-15-810) contains supplementary material, which is available to authorized users.

## Background

*Ashbya gossypii* (syn. *Eremothecium gossypii*) is a filamentous fungus that has long been known mainly as a riboflavin overproducer. Its relatively simple life cycle in the laboratory, together with the astonishing similarity of its genome with the genome of the yeast *Saccharomyces cerevisiae*, have made this fungus an attractive biological model for fungal developmental studies (reviewed in Wendland and Walther [1], Schmitz and Philippsen [2]). Based on the gene order, 91% of the 4776 annotated *A. gossypii*’s genes are syntenic and only 4% non-syntenic to *S. cerevisiae* genes [2, 3]. The remaining 5% have no homologues in *S. cerevisiae* [3].

The potential of *A. gossypii* as a host for the production of heterologous proteins has been recently investigated, through the expression of two cellulases from *Trichoderma reesei*, endoglucanase I and cellobiohydrolase I, and an *Aspergillus niger* β-galactosidase [4, 5]. *A. gossypii* possesses the capacity to secrete heterologous enzymes to the extracellular medium and to recognize signal peptides from other organisms as secretion signals [4, 5], which is a desired property for cost-efficient downstream processing of low- and medium-value enzymes. Also, it is able to perform post-translation protein modifications, such as *N*-glycosylation, and other modifications required for biological activity and protein stability [4–6]. Compared to the closely related yeast *S. cerevisiae*, one of the fungal hosts most commonly used for the production of heterologous proteins [7, 8], *A. gossypii* seems to have the tendency to hyperglycosylate less extensively (especially its *N*-glycans), which is beneficial for the production of proteins whose properties may be adversely affected by extensive glycosylation [4, 6].

These features, combined with the availability of several tools for *A. gossypii*’s easy genetic manipulation, have raised attention to this fungus as a potential cell factory organism, which could be tailor-made to produce other metabolites and/or proteins. One possible strategy that may assist with this task is the application of Systems Biology tools. Genome-Scale Metabolic Models (GSMMs) are among the most employed tools within this field. Based on the genome of an organism and on relevant physiological data, the full set of metabolic functions is assembled in a metabolic network that can be converted to the correspondent mathematical model. Central to these models is the stoichiometric matrix which covers all the metabolites and reactions present in the metabolism of the organism. Optimization tools can then be applied to these models for different purposes, such as simulate cell growth on different media, the impact of genetic engineering strategies in the phenotype or to analyze the robustness of the network [9].

The initial element in the re-construction of a genome-scale metabolic model is to collect data from the genome annotation [10], from which the enzymatic functions putatively coded within the entire genome can be collected. Such information can be consulted in non organism-specific databases, such as NCBI [11], or organism-specific databases, such as the *Saccharomyces* Genome Database (SGD; [12]) for *S. cerevisiae* or the *Ashbya* Genome Database (AGD; [13]) for *A. gossypii*.

The first genome annotation of *A. gossypii* was published in 2004 following its sequencing process [3]. This information corresponded to an association of *A. gossypii* genes to possible homologues, based on sequence similarity data. Subsequently, AGD came as a comprehensive online database presenting different information for *A. gossypii* genes based on sequence similarity and synteny degree with closely-related organisms [13]. However, despite presenting Gene Ontology (GO) data for most of *A. gossypii* genes, the information existent on AGD fails to provide a clear enzymatic functional annotation for these genes, which is usually translated in the form of EC numbers. Additionally, the genomic information for a specific microorganism is constantly evolving, as an outcome of the continuous development of bioinformatics tools, as well as the generation of new experimental data, which may allow the discovery of new proteins and genes [14] or change the information reported about them. For this reason, a process of genome re-annotation may represent a critical element to obtain reliable and updated information for a given organism. According to Ouzounis and Karp [15], an average of 7% of the genes are assigned with new functions in a re-annotation process, which represents a considerable amount of new information generated on these processes.

The functional re-annotation of an entire genome can be a very time consuming process, according to the size of the genome and the criteria applied. Possibly for this reason, only few genome-wide re-annotations were reported until now, including for *Campylobacter jejuni* NCTC11168 [16], *Mycobacterium tuberculosis* H37Rv [17], *Escherichia coli* [18], *Bacillus subtilis* [19], *Arabidopsis thaliana* [20] and, more recently, *Kluyveromyces lactis* [14]. To increase the speed of the process, some computational tools can be used to facilitate this task [21]. However, the probability of an error increases as manual curation contribution is smaller.

Here we describe a thoroughly curated genome-wide enzymatic functional re-annotation for *A. gossypii* and the first membrane transport proteins annotation for this organism. With the aim of getting further insights into *A. gossypii*’s metabolism, a comparative analysis was conducted between the metabolic capabilities of *A. gossypii* and those of *S. cerevisiae* and *K. lactis,* as these are organisms phylogenetically close to *A. gossypii* [22] and for which there is updated information available in the literature or in databases ([14]; SGD).

## Methods

### Re-annotation sources

**Source A: UniProtKB/Swiss-Prot** [23, 24] refers to a particular section of UniProtKB that presents manually annotated and reviewed information.

**Source B:*Saccharomyces*Genome Database (SGD –** [12, 25]**)** refers to information with a high degree of reliability, ensured by a team of biocurators who frequently review the information available in this platform.

**Source C: Ashbya Genome Database (AGD -** [13, 26]**)** refers to a comprehensive database containing different genomic information for *A. gossypii* based on sequence similarity and synteny degree comparatively to other organisms.

**Source D: UniProtKB/TrEMBL** [23, 24] refers to the non-reviewed section of UniprotKB.

**Source E:*merlin*** [14, 21] (available at http://www.merlin-sysbio.org/) refers to an *in-house* developed software tool that, among other features, provides homology analyses against a wide range of organisms using different tools.

### Other databases used

**Transporter Classification DataBase (TCDB -** [27, 28]**)** is a database that details a classification system for membrane transporter proteins, the Transporter Classification (TC) system.

**Kyoto Encyclopedia of Genes and Genomes (KEGG -** [29, 30]**)** is a database presenting information of the biological systems on different levels, ranging from organism related information, such as genome annotations, to enzymes specifications or metabolites information.

**BRENDA** [31, 32] is a database that gathers a very wide range of enzymes information mostly based on experimental data.

**PEDANT 3** [33, 34] is a database that presents several automatic analyses for a high amount of protein sequences. These analyses are performed based in several bioinformatics tools, which allow a wide characterization of a given protein.

### Pipeline for the functional re-annotation of the *A. gossypii*’s genome

**Figure 1:**
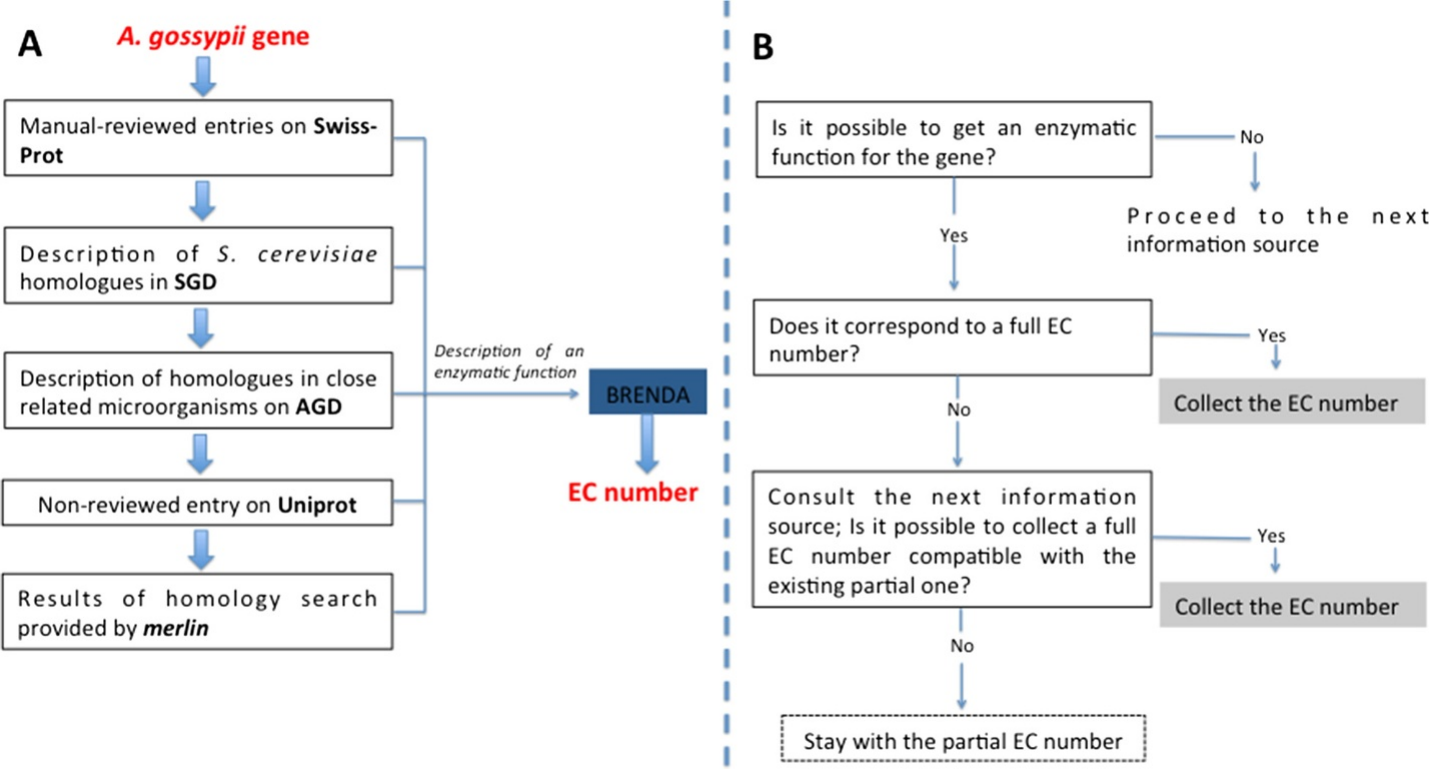
**Schematic representation of the re-annotation pipeline. (A)** order for the utilization of the different information sources; **(B)** decision-making algorithm to attribute the EC number.

The *A. gossypii*’s ATCC 10895 genome was retrieved from NCBI [11] in the form of FASTA files and loaded into *merlin* [14, 21]. For each gene, this tool performed remote Basic Local Alignment (BLAST; [35]) and Hidden Markov Models (HMMs; [36]) searches against NCBI databases (all non-redundant GenBankCDS) to find possible homologues in other species. BLAST searches were conducted using the program blastp, the matrices BLOSUM62 and PAM30, and a maximum e-value of 1E-30. Both searches were restricted to a maximum of 100 homologues. Based on the taxonomy of the organism and the number of similar hits, a confidence score (C.S.) is calculated to each possible homologue (for a detailed description see Dias *et al.* [21]). For this purpose, a α value of 0.3 was adopted on this case. If at least one possible homologue is found for a given gene (independently of the C.S.) that presents, at least, one EC number associated, that gene is considered a putative metabolic gene and the re-annotation pipeline is applied (Figure 1).

The re-annotation pipeline consists in a systematic manual process to find and collect information from different sources, enabling the assignment of one or multiple enzymatic functions to a given putative metabolic gene. Depending on the used source, the function(s) assigned to each gene had different levels of reliability. The most reliable information was retrieved from UniProtKB/Swiss-Prot, as this database contains manually curated and analyzed data. The total number of entries obtained through this source was, however, considerably small comparatively with other sources and insufficient to cover the entire genome of *A. gossypii*. To surpass this limitation, the current pipeline defines the utilization of five different information sources (Figure 1A). The utilization of these sources was defined by a decision-making structure that aimed at promoting, for every re-annotated gene, the utilization of the source with the highest reliability. To enable the collection of the most complete information possible, different databases were consulted when trying to collect a full EC number for the metabolic genes (Figure 1B).

A detailed description of the re-annotation pipeline is provided on Additional file 1.

### Annotation of membrane transporter proteins

In addition to the enzymatic functional re-annotation, membrane transporters were also sought in the *A. gossypii’*s genome. For that purpose, a recently *in-house* developed tool was employed, which allows the identification and characterization of the membrane transporter systems coded within a specific genome [37]. This tool enables to define in detail the specific transport reaction(s) that may be associated to a given gene, including the transported metabolites, reversibility, co-factor/energy usage, location on the cell, etc.. In this work, the tool was only employed to perform the initial phase of the transporter systems annotation, which consists on the assignment of a TC (Transporter Classification) number (or more than one) to putative membrane transporter-coding genes.

In an initial step, transporter candidate genes (TCGs) were identified applying a TMHMM (TransMembrane prediction using Hidden Markov Models) search over the entire genome [38], which allows predicting transmembrane helices within the several proteins coded by the genome. The presence of one putative transmembrane helice classifies the gene as a TCG, making it eligible for the second step of this process. Here, SW (Smith-Waterman) local alignments [39] are performed over the entire set of TCGs against the TCDB [27]. These alignments allow the identification of genome-coded proteins with a strong sequence similarity to TCDB entries, considering a specific similarity threshold. The standard similarity threshold is set on 10% of identity, but it can be reduced through the application of a heuristic method that considers the number of transmembrane helices initially identified. Based on the sequence similarities found for each gene, one (or multiple) TC number could be assigned to that gene. A detailed description of this tool is provided by Dias [37].

### Classification of a gene or an enzyme into a pathway

To classify a gene regarding its metabolic role in the cell, the KEGG’s internal classification system for pathways was used, taking either the classification for the gene or, if not available, the classification for the EC numbers associated to that gene. For enzyme classification, a similar strategy was adopted, consulting firstly the pathways classification on the KEGG’s page of the enzyme and, if not available, the classification provided by KEGG for associated genes.

An additional classification system was still used for the enzymes and genes with no pathway(s) assigned from KEGG, based on the FunCat nomenclature [40]. For that, the biological functions assigned to the genes that were associated to a given enzyme were retrieved from the PEDANT 3 database [33].

## Results and discussion

In this work, two main annotation processes were performed: a whole-genome functional metabolic re-annotation and the first annotation of membrane transport proteins for the fungus *A. gossypii*.

After applying the functional re-annotation pipeline described in the *Methods* section, a total of 847 genes were annotated with an EC number. Among these, 777 genes were associated with only one EC number (monofunctional genes) and 70 genes were annotated with more than one: 52 genes with 2 ECs, 11 genes with 3, 4 genes with 4, 2 genes with 5, and 1 gene with 7. Regarding the multifunctional genes, 22 presented EC numbers from different enzymatic families. One of these cases was the gene AGOS_AFR703W, which by homology with *HIS4* from *S. cerevisiae* was annotated as one oxidoreductase (1.1.1.23) and two hydrolases (3.5.4.19, 3.6.1.31), all involved in histidine biosynthesis. Another class of genes was observed that may help to understand some of the events associated to the separation of *S. cerevisiae* and *A. gossypii*. According to Brachat *et al.* [41], one of the consequences of those events was the distribution of multiple functions over different genes that were before in a multifunctional gene, which constitutes the sub-functionalization model of evolutionary divergence of duplicated genes. One of these cases is possibly AGOS_AFR234W presenting two homologues in *S. cerevisiae* with distinct functions. According to SGD, YGL224C (*SDT1*) codes for a pyrimidine nucleotidase while YER037W (*PHM8*) codes for a lysophosphatidic acid phosphatase.

It is worth noting that full EC numbers were given to 92.0% of the annotated genes, which constitutes an advantage in the context of a metabolic model reconstruction process. Partial EC numbers cannot be directly used on this process as they represent non-specific information, impairing the elaboration of a clear biochemical reactions set.

The distribution of the annotated genes by enzymatic family, depicted in Table 1, was considerably heterogeneous. Transferases was clearly the enzymatic family with the highest representation among the annotated genes, with 34.8% of the genes. That was followed by Hydrolases, Oxidoreductases, Ligases, Lyases and Isomerases.

**Table 1:** **Distribution of re-annotated genes over the different enzymatic families**

Enzymatic family	Number of genes annotated	% Annotated genes
Oxidoreductases (1.-)	195	23.0
Transferases (2.-)	295	34.8
Hydrolases (3.-)	221	26.1
Lyases (4.-)	65	7.7
Isomerases (5.-)	26	3.1
Ligases (6.-)	72	8.5

Regarding the annotation of membrane transport proteins, the first reported to date for *A. gossypii*, a total of 265 genes were annotated with one or multiple TC number(s), belonging to one of the seven families of membrane transport proteins described on TCDB. The distribution of genes over the different transporter families (Table 2) was compared to the one reported by Dias *et al.* [14] for *K. lactis* and Resende *et al*. [42] for *H. pylori*, which were obtained using the same tool [37]. From this comparison it was possible to observe that for these three different microorganisms, the transporter class 1.- 2.- and 3.- presented the highest number of associated genes, suggesting an important role of these type of transporters on the cell.

**Table 2:** **Distribution of annotated genes over the different membrane transport proteins classes**

Transporter protein class	Number of annotated genes	% Annotated genes	% Annotated genes – ***K. lactis***	% Annotated genes – ***H. pylori***
1. Channels/pores	35	13.2	9.6	13.7
2. Electrochemical potential-driven transporters	141	53.2	65.4	35.8
3. Primary active transporters	80	30.2	15.3	43.5
4. Group translocators	0	--	0.3	0.7
5. Transmembrane electron carriers	3	1.1	--	--
8. Accessory factors involved in transport	0	--	1.3	--
9. Incompletely characterized transport systems	21	7.9	8.0	6.1

### Characterization of the re-annotation sources

The enzymatic functional re-annotation performed in this work was based on different sources consulted on a top-to-bottom way in terms of reliability. Some types of information, like those originated from Class A (see Re-annotation sources in the section Methods), have a high degree of confidence as they were obtained from manually reviewed and curated sources. Other classes, however, provided information not manually curated and, therefore, less reliable. By evaluating the type of data employed on the re-annotation process, the reliability of the re-annotation results could be assessed.

**Figure 2:**
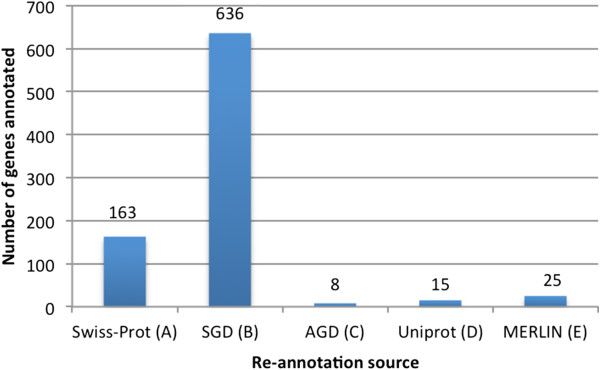
**Utilization of different information sources in the re-annotation process. (A)** manually curated data from Swiss-Prot; **(B)** manually curated data from SGD; **(C)** comprehensive database of *A. gossypii*’s genome (AGD); **(D)** non-reviewed data from Uniprot; **(E)** homology analysis data provided by *merlin*.

As can be observed in Figure 2, the main source used in the re-annotation process was the SGD, contributing for 75% of the re-annotated genes. Although this source does not provide a direct functional annotation, as Swiss-Prot does, but instead an annotation by homology, it still presents a high level of reliability, since the information retrieved from SGD is manually curated and analyzed. With a much lower, but still significant number of genes, Swiss-Prot contributed for the re-annotation of 19% of the re-annotated genes. The contribution of these two highly-reliable sources for the re-annotation of nearly 94% of the genes supports a consistent re-annotation process.

### Other available annotations for *A. gossypii*

The first complete annotation of the *A. gossypii*’s genome was publicly released in 2004 [3]. At the time, 4718 genes were annotated as protein-coding genes, but since then new protein-coding loci have been identified and the initial annotation improved. In 2007, Gattiker *et al.* [13] released the AGD 3.0, which included updated DNA annotation and microarray RNA expression data for *A. gossypii*. Subsequently, revised annotations of the reported ORFs in *A. gossypii* have been regularly updated in the NCBI GenBank database, with the latest version dating from August of 2012.

The current work provides the first manually curated whole-genome annotation of *A. gossypii*’s metabolic functions. This corresponds to the first set of curated data that directly attributes an EC number to each putative metabolic gene on *A. gossypii*. Different databases, such as BRENDA or Uniprot, provide some degree of annotation; however, they are limited to a small number of genes, insufficient for wider approaches, such as for a genome-scale metabolic model reconstruction. On the other hand, KEGG presents a very complete annotation and can, thus, serve for comparison purposes with our re-annotation. KEGG is a complex database covering multiple levels of the biological systems (genes, pathways and compounds). In what concerns gene annotation, a wide range of organisms are covered, from entire to partial genomes. For the majority of the cases, this information is, however, generated automatically from GenBank data with computational tools [30]. KEGG provides EC number information for 1111 *A. gossypii*’s genes, 264 more genes than our annotation (847). A total of 668 genes are present in both annotations, and 443 and 179 genes were exclusively annotated by KEGG and the current re-annotation, respectively. Among the set of 668 common genes, 95 presented annotations that did not match. These 95 genes were analyzed and classified into four different classes (I, II, III and IV) according to the reason why the two annotations were different.

#### Mismatches in gene annotation

Class I, which includes 35 genes, refers to the cases where the current re-annotation allowed to complete or increase the information relatively to a partial EC number annotated by KEGG. For example, the KEGG’s annotation for AGOS_ADR132W is the partial EC number 1.-.-.-, while our annotation for this gene is 1.16.3.1. This increase in the EC number information level may correspond to an important contribute on a posterior metabolic model reconstruction process.

Class II, with 17 genes, corresponds to the cases for which the current annotation added at least one new EC number comparatively with the KEGG’s annotation. This increase in the information provided by our annotation includes not only enzymatic functions that were already present in the KEGG annotation but allocated to another gene, but also new EC numbers that were not associated in KEGG to any *A. gossypii* gene. As it can be seen in Table 3, among the 25 additional EC numbers associated to genes of class II, 22 were not present in the overall KEGG annotation for *A. gossypii*, thus representing additional enzymatic functions.

**Table 3:** **Genes with additional enzymatic functions on the current annotation when compared with KEGG’s annotation (Class II)**

Gene	EC number	Product	KEGG ^1^
AGOS_ABR229C	1.1.1.9	D-xylulose reductase	No
AGOS_ACR211W	4.1.1.43	Phenylpyruvate decarboxylase	No
AGOS_ACR211W	4.1.1.74	Indolepyruvate decarboxylase	No
AGOS_ADL071C	3.1.3.68	2-deoxyglucose-6-phosphatase	Yes
AGOS_ADL287C	1.5.1.30	Flavin reductase (NADPH)	No
AGOS_ADL287C	1.5.1.39	FMN reductase (NAD(P)H)	No
AGOS_ADR115W	2.1.1.64	3-demethylubiquinol 3-O-methyltransferase	No
AGOS_ADR262C	1.6.5.3	NADH:ubiquinone reductase (H(+) translocating)	No
AGOS_AEL091C	5.4.99.28	tRNA pseudouridine32 synthase	No
AGOS_AEL091C	3.5.4.26	Diaminohydroxyphosphoribosylaminopyrimidine deaminase	No
AGOS_AEL161W	4.2.1.22	Cystathionine beta-synthase	Yes
AGOS_AEL301W	3.6.3.35	Manganese-transporting ATPase	No
AGOS_AER085C	1.3.1.10	enoyl acyl-carrier-protein reductase	No
AGOS_AER085C	2.3.1.38	[Acyl-carrier-protein] S-acetyltransferase	No
AGOS_AER085C	2.3.1.39	[Acyl-carrier-protein] S-malonyltransferase	Yes
AGOS_AER085C	4.2.1.59	3-hydroxyacyl-[acyl-carrier-protein] dehydratase	No
AGOS_AER085C	1.3.1.9	enoyl-[acyl-carrier-protein] reductase (NADH)	No
AGOS_AER085C	3.1.2.14	oleoyl-[acyl-carrier-protein] hydrolase	No
AGOS_AER401W	1.1.1.6	Glycerol dehydrogenase	No
AGOS_AER401W	1.1.1.72	Glycerol dehydrogenase (NADP(+))	No
AGOS_AFR369W	2.7.4.22	UMP kinase	No
AGOS_AFR548C	2.6.1.39	2-aminoadipate transaminase	No
AGOS_AGL199C	2.7.1.82	Ethanolamine kinase	No
AGOS_AGR002W	2.3.3.5	2-methylcitrate synthase	No
AGOS_AGR305W	3.5.4.26	Diaminohydroxyphosphoribosylaminopyrimidine deaminase	No
AGOS_AGR344W	2.7.8.1	Ethanolaminephosphotransferase	No

^1^refers to the presence of the specific EC number on KEGG’s annotation.

With 25 cases, class III presents those genes for which the two annotations reported different EC numbers, belonging either to the same enzymatic family or to distinct enzymatic families. One example is AGOS_ACL044W, annotated in KEGG with the EC number 1.2.1.3 and in the current annotation with 1.2.1.5. Despite different, these EC numbers are both associated to oxi-redox reactions. A different example is AGOS_ADR323C, annotated in KEGG as an Isomerase (5.4.2.1), but as a Hydrolase (3.1.3.37) in the present annotation.

Finally, with 18 genes, class IV includes the cases that were re-annotated in this work with levels of information lower than those available in KEGG. All of these genes were manually revised one additional time and no evidence was found that supported the extra information on KEGG.

#### Genes exclusively annotated by KEGG

The 443 genes that were exclusively annotated by KEGG were analyzed to understand their exclusion from the current annotation. KEGG provides an internal classification into pathways for some of its genes/enzymes. Based on this information (see a detailed description on the section Methods) we were able to classify 323 of the 443 genes. The remaining genes were analyzed and classified using the FunCat system (*c.f.* the section Methods for a detailed description and the Additional file 2), but are not discussed in this work, as they refer to what is considered here as non-metabolically relevant functions. Most of the genes with an available classification from KEGG were also not associated to metabolically relevant functions, but instead to pathways associated with cell maintenance and signaling, cell cycle (*e.g.* mitosis and meiosis), maintenance and modification of nucleic acids or modification of proteins (ubiquitin-related or proteases) (*c.f.* Additional file 2). Although these are all important processes for the cell, they are not directly involved in the main metabolic processes leading to the production of relevant compounds or biomass. At most, some of these functions (like those of protein kinases) may indirectly affect the metabolism of the cell, but their influence cannot be clearly stated in the re-construction of a stoichiometric metabolic model [43].

**Figure 3:**
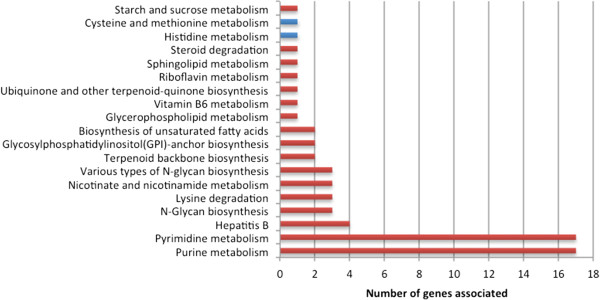
**Distribution of genes exclusively annotated by KEGG over different metabolic pathways: blue bars represent metabolically relevant functions; red bars represent non-metabolically relevant functions.**

The remaining genes, included by KEGG in putative metabolically relevant pathways (Figure 3) were distributed over a wide number of pathways. However, with the exception of only two genes (AGOS_AFR115W; AGOS_AFL046W), all of the genes were once again associated to non-metabolically relevant functions, and thus cannot be introduced in the reconstruction of a metabolic model. One of these cases corresponds to DNA-directed RNA polymerase (2.7.7.6) and DNA-directed DNA polymerase (2.7.7.7) (Purine and Pyrimidine metabolism).

#### Genes exclusively annotated by the current re-annotation

**Figure 4:**
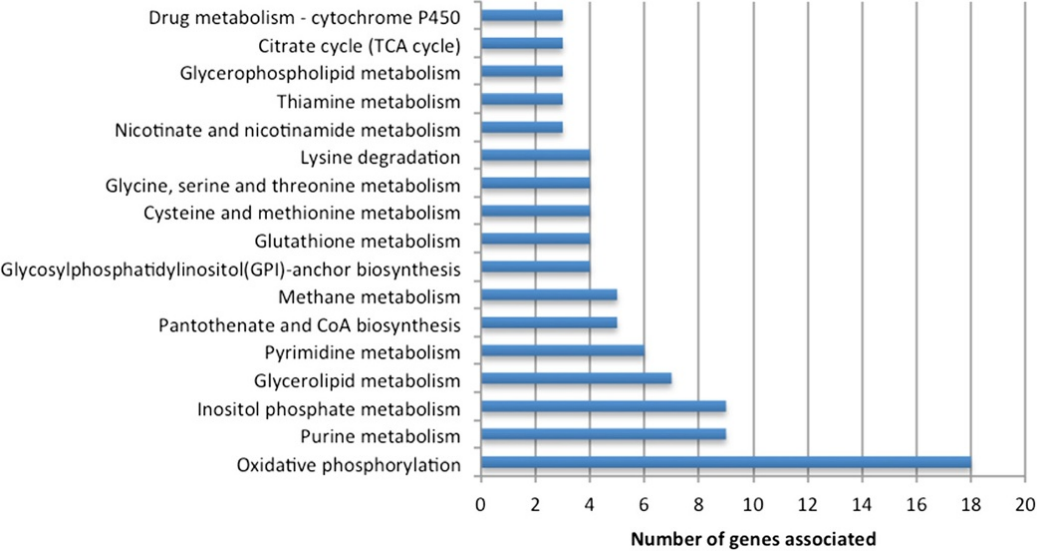
**Distribution of the genes exclusively annotated by the current re-annotation over the metabolic pathways.** Only the pathways with the highest number of associations are shown.

A total of 179 genes were exclusively annotated by the current re-annotation. The complete list of pathways associated to this set of genes is considerably extensive (*c.f.* Additional file 2), covering a wide portion of the *A. gossypii* metabolism. Figure 4 shows the metabolic pathways with the highest number of associations among the analyzed set of genes, where oxidative phosphorylation clearly shows up as the pathway with the highest number. After inspecting some of these genes, it was found that in many cases a descriptive annotation was available in KEGG, even though no EC number was provided. Thus, some of the genes presented on Figure 4 are not completely absent from the KEGG annotation.

### Metabolic capabilities of *A. gossypii*and comparison with *S. cerevisiae*and *K. lactis*

Two widely studied features of the fungus *A. gossypii* are its polarized filamentous growth and its natural riboflavin over-producing capacity. While the first has been mostly associated to regulatory mechanisms [44], the second can also be associated to the metabolic particularities of this organism. The high level of gene homology and gene order conservation between *A. gossypii* and the yeast *S. cerevisiae* has been well established, with only 5% of the *A. gossypii* annotated genes having no homologues in *S. cerevisiae* [3]. Despite the close phylogenetic relationship between these organisms, depicted in many works [22, 45, 46], contrary to *S. cerevisiae*, *A. gossypii* is a pre-WGD organism, as *K. lactis*. Thus, the re-annotation performed in this work for *A. gossypii* was compared with that available in SGD (available for download in the section *Curated Data*) for *S. cerevisiae* and the one made by Dias *et al.* [14] for *K. lactis*.

#### Enzymatic functions exclusively found in A. gossypii

SGD is probably the best resource currently available for researchers working with *S. cerevisiae*, providing manually curated information. For an individual gene it is possible to consult a description of its function(s), from which, in many cases, an associated EC number may be retrieved. This platform also provides aggregated data of the overall genome (*available for download*), which was used on this work for comparison with the *A. gossypii* re-annotation. Given the discrepancies often found in both aggregated and non-aggregated data, both sources were used for comparison with our annotation. This comparison revealed only 13 exclusive EC numbers in our re-annotation. These exclusive EC numbers were associated to a wide range of metabolic pathways (Table 4), including the metabolism of different amino acids, lipids and sugars.

**Table 4:** **Enzymatic functions exclusively found in our re-annotation comparatively to** ***S. cerevisiae*** **(functions that were also found exclusively in our re-annotation comparatively to** ***K. lactis*** **are in italic; for each EC number the information source that was used to collect it is presented)**

EC number	Enzyme	Reaction(s)	Information source
1.13.99.1	Inositol oxygenase	myo-inositol + O_2_ = D-glucuronate + H_2_O	Uniprot
*1.14.11.1*	Gamma-butyrobetaine dioxygenase	4-trimethylammoniobutanoate + 2-oxoglutarate + O_2_ = 3-hydroxy-4-trimethylammoniobutanoate + succinate + CO_2_	*merlin*
1.14.11.8	Trimethyllysine dioxygenase	N6,N6,N6-trimethyl-L-lysine + 2-oxoglutarate + O_2_ = 3-hydroxy-N6,N6,N6-trimethyl-L-lysine + succinate + CO_2_	Uniprot
1.14.19.4	Δ8-fatty-acid desaturase	phytosphinganine + reduced acceptor + O_2_ = Delta8-phytosphingenine + acceptor + 2 H_2_O	*merlin*
1.14.19.6	Δ12-fatty-acid desaturase	acyl-CoA + reduced acceptor + O_2_ = Delta12-acyl-CoA + acceptor + 2 H_2_O	*merlin*
*1.4.3.21*	Primary-amine oxidase	RCH_2_NH_2_ + H_2_O + O_2_ = RCHO + NH_3_ + H_2_O_2_	Uniprot
2.1.1.79	Cyclopropane synthetase	S-adenosyl-L-methionine + phospholipid olefinic fatty acid = S-adenosyl-L-homocysteine + phospholipid cyclopropane fatty acid	*merlin*
2.4.1.80	Ceramide glucosyltransferase	UDP-glucose + N-acylsphingosine = UDP + D-glucosyl-N-acylsphingosine	*merlin*
*3.2.1.96*	Endo-beta-N-acetylglucosaminidase	Endohydrolysis of the N,N'-diacetylchitobiosyl unit in high-mannose glycopeptides and glycoproteins containing the -[Man(GlcNAc)2]Asn- structure.	Uniprot
3.5.2.2	Dihydropyrimidinase	5,6-dihydrouracil + H_2_O = 3-ureidopropanoate	*merlin*
3.5.3.11	Agmatinase	agmatine + H_2_O = putrescine + urea	AGD
*3.5.99.6*	Glucosamine-6-phosphate deaminase	D-glucosamine 6-phosphate + H_2_O = D-fructose 6-phosphate + NH_3_	AGD
*4.6.1.13*	Phosphatidylinositol diacylglycerol-lyase	1-phosphatidyl-1D-myo-inositol = 1D-myo-inositol 1,2-cyclic phosphate + 1,2-diacyl-sn-glycerol	*merlin*

**Figure 5:**
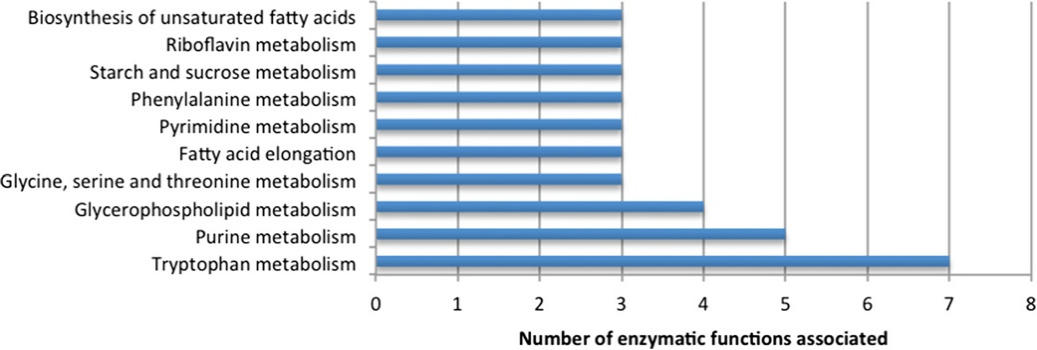
**Distribution of the enzymes exclusively found in** ***A. gossypii*** **comparatively to** ***K. lactis*** **over different metabolic pathways.**

Our re-annotation was also compared with the one reported by Dias *et al.* [14] for *K. lactis,* with 90 enzymatic functions found exclusively in *A. gossypii.* Among the pathways with the highest number of exclusive enzymes associated (Figure 5), it was possible to find carbon source-related pathways, such as the starch and sucrose metabolism, amino acid-related pathways, such as those associated to phenylalanine, glycine, serine, threonine and tryptophan, several pathways related to lipids metabolism, the pathways of purine and pyrimidine metabolism, and also the pathway of riboflavin metabolism.

From the number of exclusive functions associated to each pathway it is not possible to predict the true extension of the possible metabolic differences existent between these species, as they strongly depend of the genomic context (total functions coded within the genome). However, significant differences between the metabolome of these species may be expected in some cases, as some exclusive enzymes are associated to important metabolic pathways (*e.g.* amino acids and lipids metabolism). In other cases, the presence of these exclusive functions may only mean an alternative reactive process. One example is 4-α-glucanotransferase (2.4.1.25), exclusively found in *A. gossypii* when compared to *K. lactis*. According to KEGG, this enzyme only provides an alternative way to convert maltose into α-D-glucose, which is achieved in *K. lactis* by alpha-glucosidase (3.2.1.20).

Among the enzymatic functions exclusively found in *A. gossypii* comparatively to both *S. cerevisiae* and *K. lactis* (italicized in Table 4), there is an Endo-β-N-acetylglucosaminidase (3.2.1.96), which is involved in the hydrolysis of the N,N'-diacetylchitobiosyl unit in high-mannose glycopeptides and glycoproteins containing the -[ManGlcNAc_2_]Asn- structure. According to KEGG’s metabolic maps, this is the only route allowing the degradation of this type of glycan, therefore corresponding to an additional metabolic capacity comparatively to *S. cerevisiae* and *K. lactis*. This enzymatic activity has a broad presence reported in eukaryotes and some experimental evidences point to the existence of this activity in *A. gossypii* as well [6]. Consistently with our results, this enzymatic activity has not been detected in *S. cerevisiae* [47].

Another exclusive metabolic capacity in *A. gossypii* may be granted by a primary-amine oxidase (1.4.3.21), which allows the oxidation of a wide range of primary amines such as tyramine, phenethylamine or dopamine, thus influencing the metabolism of several amino acids (glycine, serine, threonine, tyrosine, phenylalanine). Another case corresponds to the presence of glucosamine-6-phosphate deaminase (3.5.99.6), which enables the utilization of chitin as carbon source. Opposing its absence from *S. cerevisiae* [48] and *K. lactis*, the AER268W gene from *A. gossypii* has reported similarities in AGD to SPCC16C4.10 from *Schizosaccharomyces pombe*, which is associated to the referred enzyme. This phenotype was experimentally confirmed by a positive growth of *A. gossypii* on D-glucosamine (a product of chitin degradation) as sole carbon source (*data not shown*), which requires the presence of the referred enzyme.

Associated to lysine degradation, γ-butyrobetaine dioxygenase (1.14.11.1) was another enzymatic function also found exclusively in *A. gossypii*. γ-butyrobetaine dioxygenase and trimethyllysine dioxygenase (1.14.11.8) are directly involved in the hydrolysis of protein-lysine and the final production of carnitine, a capacity that seems to be absent in *S. cerevisiae* [49].

No exclusive enzymes were found directly associated to the riboflavin metabolism that could explain the *A. gossypii* over-producing capacity for this compound. This emphasizes that, although the presence of genes is important, their regulation is often determinant, as was previously known for riboflavin biosynthesis [50, 51]. It should be reminded in this context that the simple presence of a gene with a given function does not mean the manifestation of the associated phenotype, which also reports to an important limitation of stoichiometric metabolic models. Also, a good example in this context is the work of Prinz *et al*. [44], who reported the role of regulatory mechanisms over the 26S proteasome as a determinant of filamentous form-growth for a diploid budding-yeast.

When comparing only with *S. cerevisiae*, the exclusive presence of an inositol oxygenase (1.13.99.1) in *A. gossypii* may allow the production of glucuronic acid from myo-inositol, which is one of the few catabolic pathways for this compound. Beyond that, myo-inositol was reported as an important factor on riboflavin production through regulatory mechanisms [52]. Therefore, the presence of the referred enzyme could eventually influence the production of riboflavin. Also related to inositol metabolism is phosphatidylinositol diacylglycerol-lyase (4.6.1.13), involved on the hydrolysis of 1-phosphatidyl-D-myo-inositol to 1D-myo-inositol 1,2-cyclic phosphate and diacylglycerol. Like myo-inositol, diacylglycerol is a key element in several cellular signal transduction pathways [53]. Therefore, an alteration on its intracellular pool may lead to some differences on cellular processes associated to these mechanisms.

Δ8-fatty acid desaturase (1.14.19.4) and Δ12-fatty acid desaturase (1.14.19.6) play important roles on the production of unsaturated fatty acids. Their absence in *S. cerevisiae* therefore confirms Δ9-fatty acid desaturase as the only fatty acid desaturase in *S. cerevisiae* [54] and its incapacity to produce PUFAs (poly-unsatturated fatty acids) [55]. The presence of these three enzymes in *A. gossypii*, on the other hand, suggests that it may be able to produce this class of compounds, as was already reported by Stahmann *et al*. [56].

Dihydropyrimidinase (3.5.2.2), which was associated by *merlin* to the *A. gossypii* ACR027C gene, belongs to a strict group of enzymes associated to the reductive catabolism of pyrimidines, enabling the growth on uracil, dihydrouracil, beta-ureidopropionate or beta-alanine as sole nitrogen source. Its absence from *S. cerevisiae* was supported by Andersen [57], who verified the incapacity of *S. cerevisiae* to grow at any of the referred substrates. Additionally, the author suggested that the capacity to use uracil, dihydrouracil or beta-ureidopropionate as sole nitrogen sources might have been lost at the time of the yeast genome duplication. This could indicate that *A. gossypii* (pre-WGD) may indeed present this metabolic capacity, similarly to what the author observed for *K. lactis* (pre-WGD).

Equally important is the capacity to produce cerebroside, a common membrane lipid present in fungi, which is generated by the action of ceramide glucosyltransferase (2.4.1.80). Contrarily to several yeasts, such as *Saccharomyces kluyveri*, *Zygosaccharomyces cidri*, *Zygosaccharomyces fermentati*, *K. lactis*, *Kluyveromyces thermotolerans* or *Kluyveromyces walti*, *S. cerevisiae* lacks this enzyme [58]. The presence of this enzyme in *A. gossypii* can thus lead to some differences in the composition of the cell membrane comparatively to *S. cerevisiae*.

A more peculiar case corresponds to the exclusive presence of cyclopropane synthetase (2.1.1.79), which catalyzes the production of cyclopropane fatty acids (CFAs). This enzymatic function was associated to AFR735W using *merlin* (with a 68% identity); however, no reports were found in the literature for the presence of this enzyme in any organism closely-related to *A. gossypii,* although being present in KEGG’s annotation for *Neurospora crassa*.

Finally, the possible presence of agmatinase (3.5.3.11) activity in *A. gossypii* would represent a significant modification both on the catabolism of arginine and in the synthesis of polyamines as it provides an unusual route comparatively to what is known for the degradation of arginine [59]. Additionally, it also corresponds to a new path for the production of putrescine and subsequent synthesis of polyamine, acting as an alternative to ornithine decarboxylase (ODC), as was previously demonstrated by Klein *et al.* [60]. According to Coffino [61] this alternative pathway is however still not very clear in eukaryotes.

When comparing only to *K. lactis*, the tryptophan metabolism was clearly the pathway with the highest number of enzymes found exclusively in *A. gossypii*. It was verified that five of them are associated to the genes *BNA1, BNA2, BNA4, BNA5* and *BNA7*, all involved in the kynurenine pathway for NAD biosynthesis. This result confirms the previous findings of Li and Bao [62], who verified the absence of this route in *K. lactis*, leading to an auxotrophy for nicotinic acid.

Within purine metabolism, five enzymes were found exclusively on *A. gossypii* covering, among others, the metabolism of GTP and glyoxylate, which have a reported connection to riboflavin metabolism. GTP is one of the precursors of riboflavin biosynthesis, while the glyoxylate cycle is directly involved in the formation of riboflavin precursors when plant oils are used as sole carbon sources [63].

Some differences were also found in the metabolism of different types of lipids, namely glycerophospholipids and unsaturated fatty acids. Within the synthesis of phosphatidylethanolamines (PE), it was verified that ethanolamine-phosphotransferase (2.7.8.1) and ethanolamine kinase (2.7.1.82) activities, both coded by *EKI1*, are absent in *K. lactis*. This result indicates that PE synthesis through the Kennedy pathway may not be possible on *K. lactis*, being exclusively dependent on the CDP-DAG pathway [64]. Also, the capacity to utilize diacylglycerol (DAG) for the synthesis of phospholipids may be impaired, as the *DGK1*-coded diacylglycerol kinase (2.7.1.174) is equally absent in *K. lactis*, which would mean that cells would not be able to grow in a state where the *de novo* synthesis of fatty acids can not occur [64]. Relatively to the elongation of fatty acids and the synthesis of unsaturated fatty acids, three enzymes were also lacking in the re-annotation of Dias *et al.* [14], however, strong evidences were found for their presence in the *K. lactis* genome. Very-long-chain 3-oxoacyl-CoA reductase (1.1.1.330) was recently assigned by Swiss-Prot to KLLA0B09812g, while for the very-long-chain enoyl-CoA reductase (1.3.1.93) and enoyl-CoA hydratase (4.2.1.17) coding genes, results from pBLAST searches indicated possible homologues in this organism.

In what concerns the metabolism of amino acids, beyond tryptophan, some differences were also found for glycine, serine, threonine and phenylalanine, among others. Low-specificity L-threonine aldolase (4.1.2.48) was assigned exclusively to *A. gossypii* by homology to *GLY1* from *S. cerevisiae*. Its absence from *K. lactis* may lead to slight differences in the riboflavin biosynthesis as it is directly involved in the formation of glycine, an important precursor in the *de novo* purine biosynthesis [65]. Since it is less specific than L-threonine aldolase (4.1.2.5), the presence of this enzyme in *A. gossypii* may cause a higher formation of glycine, as more substrate for this conversion is available. Associated to the metabolism of serine, D-serine hydrolase (4.3.1.18) was not annotated on *K. lactis*, which may indicate its incapacity to convert D-serine into pyruvate. This contradicts the results from a pBLAST search indicating KLLA0C11011g from *K. lactis* (74% identity) as a putative homologue of the *S. cerevisae FSH1,* which could indicate a possible error in the re-annotation of this gene. A similar case was *ARO10*-coded phenylpyruvate decarboxylase (4.1.1.43), involved in the Ehrlich pathway of amino acids catabolism. Although absent from Dias *et al.* [14] re-annotation, pBLAST (*e-value* of 1E-30) indicated a possible homologue in that organism.

Regarding carbon metabolism, three enzymes were exclusively found in *A. gossypii* associated to the metabolism of starch and sucrose. Endopolygalacturonase (3.2.1.15) catalyzes de hydrolysis of (1- > 4)-alpha-D-galactosiduronic linkages in pectate and other galacturonans (*information retrieved from KEGG*), allowing the utilization of pectin as nutrient. This result contradicts the previous observations of Murad and Foda [66], who reported the production of this enzyme on *K. lactis* when grown on a pectin-based substrate. The other two enzymes were 4-alpha-glucanotransferase (2.4.1.25) and amylo-alpha-1,6-glucosidase (3.2.1.33), which constitute the glycogen debranching system (*GDB1*), essential for glycogen utilization as carbon source [67]. This result suggests an inability of *K. lactis* to use this substrate, or an alternative route to make it, which represents a significant metabolic trait.

As can be seen on Figure 5, three enzymes were found within the pathway of riboflavin biosynthesis as present in *A. gossypii* and not present in *K. lactis*. Flavin reductase (1.5.1.30), associated to the bifunctional *ARO2* gene from *S. cerevisiae,* is involved in the reduction of FAD to FADH_2_, the first one being one of the products from riboflavin degradation. One other enzyme, 2,5-diamino-6-(ribosylamino)-4(3H)-pyrimidinone 5'-phosphate reductase (1.1.1.302), catalyzes the second step of riboflavin biosynthesis and is critical for this route. Although not referred by Dias *et al.* [14], this enzyme was already reported in *K. lactis* [68] and was recently assigned by Swiss-Prot to the *K. lactis* KLLA0F21120g gene.

#### Enzymatic functions exclusively found in other organisms

**Figure 6:**
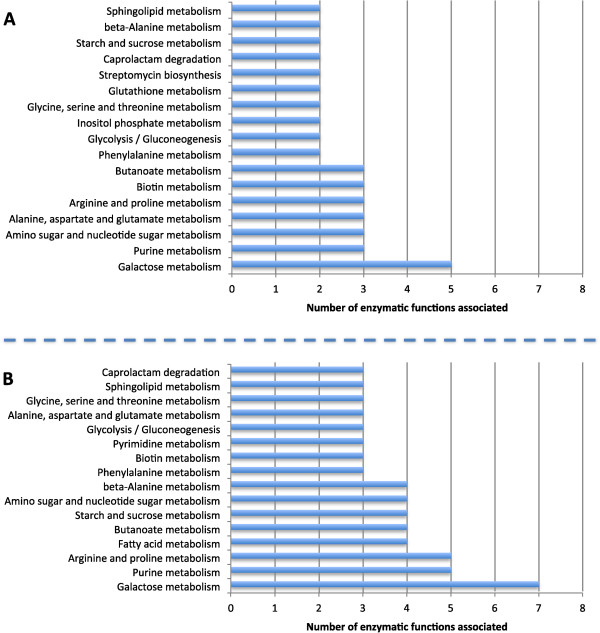
**Distribution of the enzymes exclusively found in (A)** ***S. cerevisiae*** **and (B)** ***K. lactis*** **comparatively to current re-annotation.** The enzymatic functions of *S. cerevisiae* and *K lactis* were collected from SGD and Dias et al. [14], respectively.

There were some biological functions absent in *A. gossypii* (Figure 6) that work as differentiating factors from the other two organisms.

From Figure 6 it is possible to verify that the distribution of the enzymatic functions exclusively found in *S. cerevisiae* and *K. lactis* comparatively to *A. gossypii* was quite similar, with several pathways found in common.

Similarly to what was observed for the enzymatic functions exclusively found in *A. gossypii* (Figure 5), the metabolism of purines and pyrimidines was also one of the pathways with more associated enzymatic functions absent in *A. gossypii*. The oxidation of 5-hydroxyisourate to (S)-allantoin, mediated by uric acid oxidase (1.7.3.3) and hydroxyisourate hydrolase (3.5.2.17), was one of the functions involved in these pathways that was exclusively found on *K. lactis*. A similar case was pseudouridine kinase (2.7.1.83), which has been reported for several fungus, but is absent in *S. cerevisiae* and *A. gossypii* [69]. This particular function may allow complementing a deficiency on uracil production using pseudouridine, as reported by Preumont *et al.* [69] for *E. coli*. Associated to the deamination of purines, *AAH1*-coded adenine deaminase (3.5.4.2) and *GUD1*-coded guanine deaminase (3.5.4.3) were not found in *A. gossypii*, impairing the formation of hypoxanthine and xanthine from adenine and guanine, respectively, and ultimately affecting the purine *salvage* pathway. Saint-Marc *et al.* [70] reported that a *S. cerevisiae Δaah1* strain showed a slight growth defect in the presence of adenine. According to the authors, in the presence of adenine and in the absence of Amd1p, the synthesis of IMP and GMP would take place mostly through an *AAH1, HPT1*-mediated route. The most unexpected finding corresponded, however, to the exclusive presence of β-alanine synthase (3.5.1.6) on *K. lactis*, which is directly involved in the catabolism of pyrimidines. According to LaRue and Spencer [71], most of the fungi do not present this specific route, impairing the utilization of pyrimidines (or their degradation products) as sole nitrogen sources. The KLLA0D03520g gene from *K. lactis* showed, however, a significant similarity to *PYD3* from *S. kluyveri,* which codes for a β-alanine synthase, indicating that this capability may be present on this organism as well. A final difference in the *de novo* biosynthesis of pyrimidines consists on the *URA1*-coded dihydroorotate dehydrogenase (DHOD) (1.3.98.1), involved in the *de novo* biosynthesis of pyrimidines, which is absent in *A. gossypii*. As described by Hall *et al.* [72], this seems to be an example of horizontal gene transfer from bacterial lineages, and more specifically, a case of gene displacement. *URA1* codes for a DHOD type 1, which is optimized for anaerobic conditions. According to Hall *et al.* [72], the fixation of this bacterial gene in the genome of *S. cerevisiae* may indicate that its evolution involved adaptation to anaerobic environments, providing the selective pressure to maintain the referred gene. On the other hand, *URA9*, which has a homologue in *A. gossypii* but not in *S. cerevisiae*, codes for a type 2 DHOD (1.3.5.2) and seems to be oriented for aerobic conditions, corroborating the difficulty of *A. gossypii* to grow in anaerobic conditions [72].

In terms of carbon metabolism the main differences were found associated to the utilization of lactose and galactose. In contrast to *S. cerevisiae* and *K. lactis*, four enzymes of the Leloir pathway [73] were not found in *A. gossypii*, namely the *GAL1*-coded galactokinase (2.7.1.6), *GAL7*-coded galactose 1-phosphate uridyltransferase (2.7.7.12), *GAL10*-coded UDP-galactose 4-epimerase (5.1.3.2) and *GAL10*-coded aldose 1-epimerase (5.1.3.3). This result supports the inability reported for *A. gossypii* to grow on galactose [74], as these are critical enzymes on this metabolic route. Also, β-galactosidase (3.2.1.23), which is associated to lactose utilization, was only found in *K. lactis*, confirming the exclusive presence of this important trait on this organism. β-glucosidase (3.2.1.21) was another exclusive enzyme found on this organism. This enzyme, which catalyzes the conversion of cellobiose to β-D-glucose, is directly involved in the degradation of cellulose [75], assuming a considerable importance in a biotechnological context. Both *A. gossypii* and *S. cerevisiae* present genes coding for the glucan 1,3-β-glucosidase (3.2.1.58); however, this enzyme acts on β-(1- > 3) bonds, impairing therefore cellobiose degradation. A more interesting case refers to the utilization of maltose. Although isomaltase (3.2.1.10; coded by *IMA1, IMA2, IMA3, IMA4 and IMA5*) and maltase (3.2.1.20; coded by *MAL12 and MAL32*) were absent in *A. gossypii*, some old reports suggest the capability to utilize this carbon source [76]. More specifically, Mickelson [77] reported that *A. gossypii* was able to oxidize maltose, though more slowly comparatively to glucose and sucrose. Although no homologues were detected regarding the above-mentioned maltase and isomaltase, this phenotype can have occurred through other unknown systems allowing maltose utilization, such as 4-alpha-glucanotransferase (2.4.1.25). Another possibility that should be considered is the existence of small differences between different strains of the same specie as recently exposed by Ribeiro *et al.* [78] and by Dietrich *et al.* [79].

Within nitrogen metabolism, several differences were observed over different amino acids. Specifically associated to glutamate degradation, it was observed that *GDH1* (or *GDH3*)-coded NADP^+^-dependent glutamate dehydrogenase was absent comparatively both with *S. cerevisae* and *K. lactis*, indicating that *A. gossypii* possibly does not have the glutamate dehydrogenase ammonium assimilation route [80]. Also, *GAD1*-coded glutamate decarboxylase was not present, impairing the gamma-aminobutyrate (GABA) route of glutamate degradation [81]. Involved in both alanine and glycine metabolism, *AGX1*-coded alanine:glyoxylate aminotransferase (2.6.1.44) was lacking on this organism, which may have a significant impact on riboflavin biosynthesis as it conducts one of the main routes for glycine formation on yeasts. This result confirms previous studies aiming the improvement of riboflavin production through the introduction of this route [82]. *GCV1*-coded T subunit of mitochondrial glycine decarboxylase complex was also not found in *A. gossypii*, which may suggest its incapacity to utilize glycine as sole nitrogen source [83]. Indirectly related to glycine synthesis, it was observed the exclusive presence of 2-hydroxyglutarate dehydrogenase (1.1.99.2) on *K. lactis*, which mediates the formation of 2-hydroxyglutarate from 2-oxoglutarate. Despite this absence, Albers *et al.* [84] have previously suggested that this activity may be present in *S. cerevisiae* associated to *YER081W* and *YIL074C*, which have a homologue in *A. gossypii* (*ACL032C*). Involved in the Ehrlich pathway of amino acids degradation, the absence of *ARO9*-coded tryptophan:phenylpyruvate transaminase (2.6.1.28) was verified in *A. gossypii*, confirming previous reports indicating its inability to degrade amino acids into fusel acids/fusel alcohols [85]. Relatively to sulfur-related amino acids, it was observed the lack of *SPE4*-coded spermine synthase (2.5.1.22), which is directly associated to spermine biosynthesis from S-adenosylmethionine and the final production of volatile sulfur compounds (VSC). This result is in accordance with the previous study of Hébert *et al.* [86], who reported several differences in the sulfur amino acid pathways among hemiascomycetous yeast, such as the absence of *SPE4* in *A. gossypii*.

Sphingolipid metabolism also presented a few differences among the species. The first one refers to *DPL1*-encoded sphinganine-1-phosphate lyase (4.1.2.27), which was only found in *S. cerevisiae* and *K. lactis*. According to different reports [87, 88], this gene may play a critical role in the cell response to nutrient starvation through changes on the internal concentration of sphingosine-1-phosphate. More specifically, Gottlieb *et al.* [87] observed that a *Δdpl1* strain of *S. cerevisiae* presented an unusual accumulation of phosphorylated sphingoid bases, leading to an unregulated proliferation close to the stationary phase. The other difference is related to the exclusive presence of *BDS1*-encoded arylsulfatase (3.1.6.1) in *S. cerevisiae* and *K. lactis*. Similarly to the above mentioned *URA1*, this may also represent a case of horizontal gene transfer [72], which could explain its absence from *A. gossypii*. Like *URA1*, the acquisition of this function may have derived from an environmental pressure to utilize alternative sulfur sources, which may be beneficial in a constrained environment. Hall *et al.* [72] observed a small increase on the cell growth of wild-type *S. cerevisiae* comparatively to a Δ*bds1* strain when a medium supplemented with 4-nitrocatechol sulfate was used.

Still related to the overall metabolism of lipids, the β-oxidation of fatty acids also presented some differences. Contrarily to *K. lactis*, *S. cerevisiae* and *A. gossypii* did not present the set of enzymes constituted by 1.3.8.7, 1.3.8.8 and 1.3.8.9, which is directly involved in the first step of fatty acids β-oxidation. Instead, they presented an equivalent system associated to *POX1* gene from *S. cerevisiae* and with a homologue in *A. gossypii*. The main difference between these systems lies on the type of electron transfer mechanism and intermediates. While the first system, mainly mitochondrial, uses an electron transport chain until the final receptor-oxygen-, the second one (*POX1*), which is peroxisomal, transfers instead the electrons directly to oxygen [89]. It is worth noting, however, that the presence of the first system in *K. lactis* is an unexpected finding, as fungal organisms are mainly characterized by the peroxisomal system [90].

A common reported phenotype in *A. gossypii* is its incapacity to synthesize biotin [91], confirmed on this case by the absence of *BIO2*, *BIO3* and *BIO4* coded enzymes, directly associated to this process [92], which were found in both *S. cerevisiae* and *K. lactis*. A similar result was found for *myo*-inositol, supported by the absence of *INO1*-coded myo-inositol-1-phosphate synthase (5.5.1.4). Also related to inositol biosynthesis, inositol-phosphate phosphatase (3.1.3.25) was exclusively found in *S. cerevisiae* (*INM1* and *INM2*). However, contrarily to the previous case, this function seems not to be essential, as a double mutation of *INM1* and *INM2* did not affect growth and inositol biosynthesis in *S. cerevisiae* [93].

## Conclusions

A functional re-annotation pipeline was designed and applied to the entire genome of *A. gossypii* rendering the first enzymatic functional annotation for this organism. After applying this pipeline, 847 genes were manually assigned with metabolic enzymatic functions. Additionally, the first annotation of the membrane transport proteins for this organism was also performed, which allowed to identify and classify 265 genes as potential membrane transporters. Comparatively to KEGG, this re-annotation allowed correcting the information of 95 genes of *A. gossypii*, ranging from completing EC numbers, adding new EC numbers or removing outdated information. Among these 95 genes it was possible to assign 22 new enzymatic functions that were completely absent from KEGG’s annotation for *A. gossypii,* which corresponds to a significant improvement in the knowledge of its metabolism.

Also, a comparative analysis between the set of EC numbers associated to *S. cerevisiae*, *K. lactis* and *A. gossypii* provided an overall perspective over some of the main differences among their metabolism. When compared to *S. cerevisae*, *A. gossypii* only presented 13 exclusive EC numbers. This number was considerably higher when this comparison was made with *K. lactis* (90). In both cases these EC numbers were spread into different parts of the metabolism, namely carbon and nitrogen metabolism, lipids, purine and pyrimidines, among others, with no enriched part in particular. The similar happened for the enzymatic functions that were absent in *A. gossypii* but not in *S. cerevisiae* and *K. lactis*. These last analyses found fundamental evidences for some of the well-known phenotypes of *A. gossypii* such as the inability to use lactose and galactose, and to synthesize biotin and myo-inositol, among others.

This work reports the first manual genome-wide enzymatic functional annotation for the metabolism of *A. gossypii*. As manually curated data, it represents a high quality information source, providing an increased knowledge of the *A. gossypii*’s metabolism. Additionally, the comparative study performed with the metabolism of the closely-related yeasts *S. cerevisiae* and *K. lactis* also provided relevant insights into the *A. gossypii*’s metabolism. Together with the membrane transporters annotation, this whole data set constitutes a solid and more complete platform for the development of different studies, namely in the field of fungal biology and systems biology.

### Availability of supporting data

The data sets supporting the results of this article are included within the article (and its additional files).

## Electronic supplementary material

Additional file 1: **Figure A1- Pipeline used for the metabolic functional re-annotation of the** ***A. gossypii*** **genome; Detailed description of the pipeline.** (DOCX 286 KB)

Additional file 2: **Supplement 1-Metabolic genome-wide functional re-annotation of** ***Ashbya gossypii*** **ATCC 10895; Supplement 2- Overall statistics of the functional re-annotation; Supplement 3- Comparison of the annotations for the 668 genes annotated both on KEGG and on the current re-annotation; Supplement 4- Membrane transport proteins annotation of** ***Ashbya gossypii*** **ATCC 10895; Supplement 5- Overall statistics of the transport proteins annotation; Supplement 6- Set of EC numbers of** ***S. cerevisiae*** **(SGD),** ***K. lactis*** **(Dias** ***et al.*** [14]**]) and** ***A. gossypii*** **(current re-annotation); Supplement 7- Genes annotated exclusively by KEGG and their classification into metabolic pathways; Supplement 8- Genes annotated exclusively in the current re-annotation and their classification into metabolic pathways; Supplement 9- Enzymatic functions exclusively found in** ***A. gossypii*** **comparatively to** ***S. cerevisiae*** **; Supplement 10- Enzymatic functions exclusively found in** ***A. gossypii*** **comparatively to** ***K. lactis*** **; Supplement 11- Enzymatic functions exclusively found in** ***S. cerevisiae*** **comparatively to** ***A. gossypii*** **; Supplement 12- Enzymatic functions exclusively found in** ***K. lactis*** **comparatively to** ***A. gossypii***. (XLS 551 KB)

## Abbreviations

AGD: *Ashbya* Genome Database
BLAST: Basic local alignment search tool
CFAs: Cyclopropane fatty acids
DAG: Diacylglycerol
DHOD: Dihydroorotate dehydrogenase
EC: Enzyme commission
GABA: Gamma-aminobutyrate
GO: Gene Ontology
GSMMs: Genome-scale metabolic models
HMMs: Hidden markov models
KEGG: Kyoto encyclopedia of genes and genomes
ODC: Ornithine decarboxylase
PE: Phosphatidylethanolamines
PUFAs: Poly-unsatturated fatty acids
SGD: *Saccharomyces* Genome Database
SW: Smith-Waterman
TC: Transporter classification
TCDB: Transporter classification database
TCGs: Transporter candidate genes
TMHMM: TransMembrane prediction using Hidden Markov Models
VSC: Volatile sulfur compounds
WGD: Whole genome duplication.
