# Supplementary material for: Genome-wide metabolic re-annotation of Ashbya gossypii: new insights into its metabolism through a comparative analysis with Saccharomyces cerevisiae and Kluyveromyces lactis
Source: BMC Genomics. 2014 Sep 24;15(1):810. doi: 10.1186/1471-2164-15-810 (PMC4190384; doi:10.1186/1471-2164-15-810)
Supplement: Supplementary file 1 — Additional file 1: Figure A1- Pipeline used for the metabolic functional re-annotation of the A. gossypii genome; Detailed description of the pipeline. (DOCX 286 KB) [file 12864_2014_6500_MOESM1_ESM.docx]

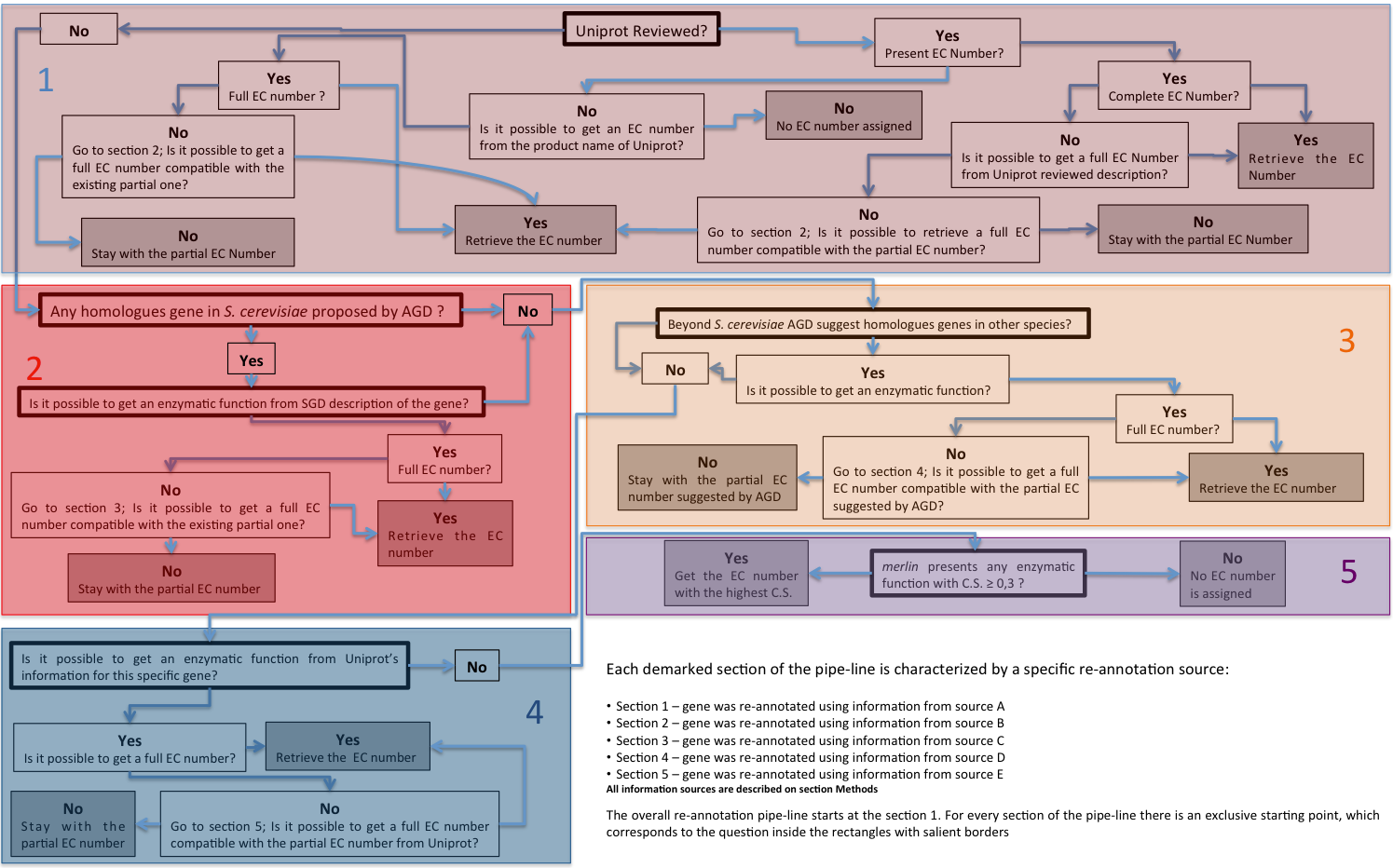


**Figure A1. Pipeline used for the metabolic functional re-annotation of the *A. gossypii* genome.**

The re-annotation pipeline designed consists in 5 distinct sections, which were followed sequentially until a stopping criteria was reached:

**1** – UniProtKB was consulted to verify which genes were manually reviewed (i.e. had a Swiss-Prot entry). Reviewed genes that presented a full EC number were immediately re-annotated with that EC number. If only a partial EC number was associated to the reviewed gene or if the gene was manually reviewed at UniProtKB but with no associated EC number, the gene’s product name was used on BRENDA to obtain a complete EC number or the following sections of the pipeline were used. The first complete EC number obtained was immediately assigned to the gene if it was compatible with the partial one available in Swiss-Prot or if no partial EC number was available. Otherwise, the gene was annotated with the partial EC number assigned to it in Swiss-Prot. If no partial EC number was associated to the gene in Swiss-Prot and a full EC number could not be obtained in any of the subsequent sections, the gene was annotated with the most informative partial EC number obtained. To the genes that were not reviewed at UniProtKB, the section 2 of the pipeline was immediately applied.

**2** – AGD (http://agd.vital-it.ch/index.html) was consulted to verify if there were any homologue(s) in *S.* *cerevisiae* to the gene under analysis. This information was updated with a BLASTp search against NCBI, with a maximum e-value of 1E-30. If so, a detailed description of that/those homologue gene(s) was consulted in SGD (http://www.yeastgenome.org/) to collect putative enzymatic functions. When a full EC number was obtained from BRENDA using this description, the gene was immediately re-annotated with that EC number if it was compatible with the existing partial one. If not, or if only a partial EC number could be retrieved, the following sections of the pipeline were used to try to obtain a complete EC number. When no *S. cerevisiae* homologue was initially suggested by AGD or from the description provided by SGD, no enzymatic function could be retrieved and, thus, the following sections of the pipeline were immediately applied to the gene under analysis.

**3** – AGD was consulted to verify if there were any homologue(s) to the gene under analysis in other species beyond *S. cerevisiae* (like *K. lactis*, *N. crassa* or *S. pombe*). This information was updated with a BLASTp search against NCBI as in the previous point. When that was the case and an enzymatic function could be retrieved, BRENDA was consulted to get the associated EC number, which was assigned to the gene only if it was compatible with the existing information. If only a partial EC number could be retrieved from that information, the following sections of the pipeline were used to try to complete that EC number. On the other hand, if no homologues beyond those from *S. cerevisiae* were indicated by AGD, or if they existed but were unable to provide an enzymatic function, the following sections of the pipeline were applied to the gene under analysis.

**4** – The “Ontologies” section from UniProtKB was consulted for the gene under analysis. When from that information an enzymatic function could be retrieved from BRENDA, that was assigned to the gene if it was compatible with the existing information. If only a partial EC number could be retrieved, the next section of the pipeline was used to try to complete that EC number. On the other hand, if from the UniProtKB description no enzymatic function could be retrieved, the last section of the pipeline was immediately applied.

**5** – The homology information initially obtained with the *merlin* software was consulted for the gene under analysis. If an enzymatic function with a C.S. (c.f. Dias *et al.* [14]) equal or higher than 0.3 was available, that was assigned to the gene when it was compatible with the existing information. For multiple EC numbers meeting this requirement, the EC number with the highest C.S. was selected.

When no EC number (partial or complete) could be retrieved in any of the sections, no EC number was assigned to that gene.
